# Tigilanol Tiglate-Induced Changes in Secretome Profiles Alter C-Met Phosphorylation and Cell Surface Protein Expression in H357 Head and Neck Cancer Cells

**DOI:** 10.3390/cells13110982

**Published:** 2024-06-05

**Authors:** Frank Dickson Antwi, Tufaha Awad, Meghan Larin, Kate Heesom, Phil Lewis, Paul Reddell, Zaruhi Poghosyan, Sharon Dewitt, Ryan Moseley, Vera Knäuper

**Affiliations:** 1 School of Dentistry, College of Biomedical and Life Sciences, Cardiff University, Cardiff CF14 4XY, UKdewitt@cardiff.ac.uk (S.D.); moseleyr@cardiff.ac.uk (R.M.); 2 Bristol Proteomics Facility, Biomedical Sciences Building, University Walk, University of Bristol, Bristol BS8 1TD, UK; 3 QBiotics Group, Yungaburra, QLD 4884, Australia; 4 School of Medicine, College of Biomedical and Life Sciences, Cardiff University, Cardiff CF14 4XN, UK

**Keywords:** cancer, secretome analysis, therapy

## Abstract

Tigilanol tiglate (TT, also known as EBC-46) is a novel, plant-derived diterpene ester possessing anticancer and wound-healing properties. Here, we show that TT-evoked PKC-dependent S^985^ phosphorylation of the tyrosine kinase MET leads to subsequent degradation of tyrosine phosphorylated p-Y^1003^ and p-Y^1234/5^ MET species. PKC inhibition with BIM-1 blocked S^985^ phosphorylation of MET and led to MET cell surface accumulation. Treatment with metalloproteinase inhibitors prevented MET-ECD release into cell culture media, which was also blocked by PKC inhibitors. Furthermore, unbiased secretome analysis, performed using TMT-technology, identified additional targets of TT-dependent release of cell surface proteins from H357 head and neck cancer cells. We confirm that the MET co-signalling receptor syndecan-1 was cleaved from the cell surface in response to TT treatment. This was accompanied by rapid cleavage of the cellular junction adhesion protein Nectin-1 and the nerve growth factor receptor NGFR^p75^/TNFR16. These findings, that TT is a novel negative regulator of protumorigenic c-MET and NGFRp^75^/TNFR16 signalling, as well as regulating Nectin-1-mediated cell adhesion, further contribute to our understanding of the mode of action and efficacy of TT in the treatment of solid tumours.

## 1. Introduction

More than 600,000 new head and neck cancers are diagnosed annually, with 5-year survival rates remaining below 50%. Unfortunately, the median survival rate of distant metastatic disease is 6 months. Thus, novel therapies targeting disease recurrence or tumour growth mechanisms are urgently needed to improve patient survival. 

**Figure 1:**
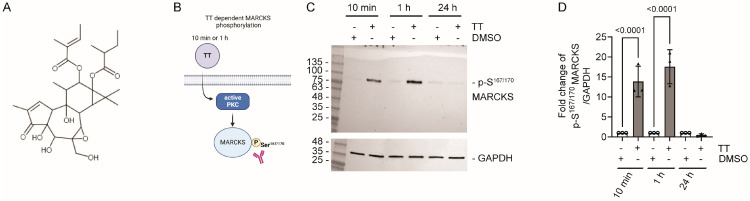
**TT (EBC-46) dependent phosphorylation of p-S^167/170^ MARCKS.** (**A**) Chemical structure of TT. (**B**) Schematic representation of experimental hypothesis prepared in Biorender. (**C**) Western blot analysis of S^167/170^ phosphorylation levels on MARCKS after 10 min, 1 h, and 24 h TT treatment. A GAPDH loading control is shown below. (**D**) Fold change of MARCKS phosphorylation on S^167/170^ in response to TT treatment (*n* = 3). One-way ANOVA with post-Tukey test is shown.

Tigilanol tiglate (TT, previously known as EBC-46, [12-tigloyl-13-(2 methylbutanoyl)-6,7-epoxy-4,5,9,12,20-hexahydroxyl-1-tigliane-3-on]) (Figure 1A) is a novel epoxytigliane isolated from the seed of the Australian rainforest plant, *Fontainea picrosperma* [1,2]. TT has been shown to possess potent anticancer properties for local treatment of a range of solid tumours in mouse models, in spontaneous tumours presenting in the veterinary clinic, and in early-phase human clinical safety and efficacy trials. TT has a multifactorial mode of action in tumour destruction that is mediated, at least in part, by activation of specific classical isoforms of protein kinase C (PKC), especially PKC-βI and -βII [1,2,3,4,5]. Following intratumoral administration, TT causes a rapid, but localised, inflammatory response, direct oncolysis of tumour cells and loss of integrity in the tumour vasculature, which in combination lead to tumour haemorrhagic necrosis and ablation [1,6]. TT also stimulates rapid re-epithelialisation of tissue deficit that forms following destruction of the treated tumours [7]. Additionally, TT has been shown to act as a small oncolytic molecule which induces immunogenic cell death. This activity enhances the response of both TT-injected and non-injected tumors to immune checkpoint blockade, which has been mechanistically linked to a caspase/gasdermin E-dependent pyroptotic pathway [6]. TT has now been registered as a veterinary pharmaceutical for treatment of canine mast cell tumours [4,8], (trade name STELFONTA^®^) by regulatory authorities in the US, Europe, United Kingdom, and Australia, and is in human Phase II clinical trials for treatment of head and neck cancers and soft tissue sarcomas [9] (Clinical Trials.Gov ID, NCT05235537; NCT05608876; NCT05755113).

TT is in the same broad chemical class as the prototype PKC-activator phorbol myristyl acetate (PMA), but with a more specific PKC activation profile and, on prolonged exposure, not resulting in loss of PKC function as occurs with PMA. The PKC pathway has attracted attention as regulating tumour suppressor function [10]. PMA is known to activate a wide range of classical and novel PKC isoforms, which subsequently cause activation of metalloproteinases from the disintegrin and metalloproteinase family (ADAMs) and lead to ectodomain shedding of numerous cell surface substrates [11,12,13,14] allowing for the detection of cancer biomarkers [11,15]. Additionally, ADAM activity regulates major signalling pathways involved in cancer progression and metastasis (reviewed in [16]). In this context, PMA-dependent serine phosphorylation of the receptor tyrosine kinase MET on S^985^ was previously observed, which led to downregulation of MET signalling responses [17]. MET is a cancer driver in solid tumours [18], and this receptor is involved in cancer recurrence signalling in response to human epidermal growth factor 2 (HER2) tyrosine kinase inhibitors [19]. Therefore, drugs targeting MET S^985^ phosphorylation, MET cell surface levels or those that produce apoptotic MET fragments are potentially desirable as anticancer therapeutics [20]. 

We used H357 tongue cancer cells, a good model of the most affected area in head and neck cancer, to study novel therapies, such as TT treatment. We set out to investigate early global changes in the secretome of H357 cells using isobaric labelling with tandem mass tag technology (TMT) to discover early TT drug responses and additionally show that TT causes PKC dependent phosphorylation of S^985^ MET and cleavage of the MET tyrosine kinase. 

## 2. Materials

TT was supplied by QBiotics Group (Yungaburra, Australia). ADAM inhibitors GW280264X (GW) and GI254023X (GI) targeting ADAM17 and ADAM10, respectively, were a kind gift from Dr Augustin Amour at GlaxoSmithKline (Stevenage, UK), and were used at 1 µM concentration. GM6001 was from Merck Life Sciences (Watford, UK) and was used at 25 µM concentration. The PKC inhibitors Gö6976 (GOE), Enzastaurin and Bisindolylmaleimide (BIM-1) were from Merck. All PKC inhibitors were used at 1 µM concentrations. 

The following antibodies were from R&D systems (Abingdon, UK): anti-PTPRU (clone 764209), anti-human ADAM17 ectodomain (clone 111633), anti-human ADAM10 (clone 163003), anti-p-Y^1003^ MET (AF4059), anti p-Y^1234/5^ MET (AF2480), anti HGFR/c-MET PE-conjugated antibody (clone 95106) and mouse IgG1 PE-conjugated antibody (clone 11711).

Anti-p-S^985^ MET antibody (PA5-64558) was from ThermoFisher Scientific (Paisley, UK). Phospho-MARCKS (p-S^167/170^) (D13E4) XP^®^ rabbit monoclonal antibody was from Cell Signaling Technology (Danvers, MA, USA). N-terminal Nectin-1 antibody (clone OTI6E11) was from Origene Technologies (Rockville, MD, USA). C-terminal MET antibody (NBP2-43641) was from Bio-Techne Ltd. (Abington, UK). Anti NGFR^p75^/TNFR16 (ab52987) rabbit monoclonal antibody was from Abcam (Cambridge, UK).

HRP-conjugated AffiniPure goat anti-mouse IgG (115-035-003) was from Jackson Immuno Research (Ely, UK). GAPDH (60004-1-Ig), HRP-conjugated AffiniPure goat anti-rabbit IgG (SA00001-2) and HRP-conjugated AffiniPure goat anti-mouse IgG (SA00001-1) were from Proteintech (Manchester, UK).

H357 head and neck cancer cells were purchased from Merck Life Sciences.

## 3. Methods

### 3.1. Tissue Culture

H357 cells were seeded at 1 × 10^6^ cells/well in six well plates and grown for 72 h in DMEM/10% FCS until they reached 60–80% confluency. Cells were washed with serum-free Opti-MEM and either pre-incubated with metalloproteinase [25 μM for GM6001; 1 μM for GW or GI] or PKC inhibitors [1 μM] or DMSO solvent control in Opti-MEM for 1 h at 37 °C. Cells were then stimulated with 1 µg/mL TT for either 10 min, 1 h or 24 h. It is indicated in each results figure whether cells were then treated with 1 μg/mL TT alone or in the presence of metalloproteinase or PKC inhibitors at above concentrations. A DMSO solvent control is included in all experiments, as well as TT treatment at 1 μg/mL for the indicated time points.

### 3.2. Western Blotting and Quantification of Proteins in Cell Lysates

Cell lysates were prepared using either Pierce RIPA-buffer supplemented with complete detergent compatible EDTA-free inhibitor cocktail (1 tablet/10 mL buffer, Roche, Burgess Hill, UK), 10 mM 1,10 phenanthroline and PhosStop (1 tablet/10 mL buffer, Roche) or 50 mM HEPES (pH 7.5), 10 mM N-ethylmaleimide, 100 µM benzamidine, 0.5% Nonidet P-40, 250 mM NaCl, 2 mM EDTA, 10% (*v*/*v*) glycerol lysis buffer supplemented with the same protease and phosphatase inhibitors. Lysate protein concentrations were determined using the BCA protein assay (Bio-Rad, Hemel Hempstead, UK) in conjunction with a BSA standard curve according to the manufacturer’s instructions. A FLUOstar Omega BMG-Labtech plate reader (Aylesbury, UK) was used to determine optical densities.

A total of 20 to 40 µg of cell lysate was diluted 1:2 using 2× reducing SDS sample buffer, boiled for 5 min and loaded either onto 4–20% mini-protean TGX precast gels (Bio-Rad) or home cast 10% or 12% SDS-PAGE gels. Proteins were separated at 100 V until the dye front reached the bottom of the gel. Gels were transferred to either PVDF or nitrocellulose membranes at 75 V for 1–2 h. Membranes were blocked with either 5% skimmed milk in TBST (PVDF) or 5% BSA in TBST (nitrocellulose; phosphoproteins) unless otherwise specified by the antibody manufacturer and incubated for 1 h at room temperature (RT). Blots were incubated with appropriate primary antibodies for 24 h at 4 °C in blocking solution. Blots were washed with TBST prior to incubation for 1 h at RT with the appropriate secondary HRP conjugated antibody, washed three time with TBST, prior to incubation in SuperSignal™ West Pico Plus chemiluminescent substrate (Thermoscientific). Blots were imaged using the iBright 1500 imaging system using smart exposure (Invitrogen, Waltham, MA, USA) or custom exposure if signal intensities were low. Blots were reprobed with GAPDH mouse monoclonal primary antibody at 1:10,000 dilution and goat anti-mouse HRP conjugated secondary antibody at 1:5000 in 5% skimmed milk. All antibodies and sources are listed in Materials.

Quantification of Western blot bands was performed using iBright analysis software by determining background corrected densities for the target bands and the appropriate GAPDH loading control for each blot. The data were exported to an Excel spreadsheet and ratios of target band/GAPDH background corrected densities calculated. Data were normalised to solvent control conditions for each experiment and the appropriate time points as follows to determine the fold change.

Fold change = (Target^Treated^/GAPDH^Treated^)/(Target^Control^/GAPDH^Control^)

Data were entered into GraphPad Prizm for graphing and statistical analysis using either one-way ANOVA with post-Tukey correction or two-tailed T-tests with Welch’s correction, as stated in the figure captions. At least three independent experiments were analysed. Figures were prepared with Adobe Illustrator and Biorender.

### 3.3. Flow Cytometry

Treated cells were washed in warm PBS prior to incubation with Gibco™ enzyme free dissociation buffer (Gibco, Fisher scientific 11530456, Waltham, MA, USA) for 10 min to detach the cells. Cells were collected, diluted with PBS in Falcon tubes, and centrifuged at 1500 rpm for 5 min. The cell pellet was resuspended into 100 µL warm PBS and transferred into a 96-well plate and centrifuged for 5 min at 1200 rpm. AmCyan live dead stain was added to the cells and incubated for 30 min at RT, prior to centrifuging for 5 min at 1200 rpm. The cell pellet was incubated with either control IgG_1_-PE or anti-hHGFR/c-MET-PE conjugated antibodies at 1 in a 100 dilution for 30 min at 4 °C. The plate was centrifuged for 5 min at 1200 rpm, supernatant removed, and fixed for 15 min at RT in 200 µL 4% formaldehyde/PBS. Following further centrifugation, the supernatant was removed from the cells and suspended in 200 µL 2% FCS/PBS and stored in the dark at 4 °C until analysis.

Compensation of spectral overlap was set up using UltraComp eBeads (Invitrogen) that were either stained with anti-hHGFR/c-MET-PE conjugated antibody or a V500-conjugated antibody (BD Biosciences, Franklin Lakes, NJ, USA; 561391) that mimics the live/dead stain. Beads were centrifuged at 1200 rpm for 3 min and the supernatant discarded. Beads were then suspended in 200 µL 2% FCS/PBS. Samples were analysed using the BD biosciences Canto II flow cytometer running FACSDiva software. Stained cells were gated, so that dead cells were excluded, and results were analysed using Flowjo 10 software. For gating strategy, see Supplemental Figure S3. The geometric mean fluorescence intensities (GMFI) were obtained and further analysed in GraphPad Prism. Data were analysed using one-way Anova with Tukey post-test.

### 3.4. ELISA for Quantifying MET-ECD and Syndecan-1 ECD

Human MET DuoSet^®^ ELISA kit (DY358) and human syndecan-1 DuoSet^®^ ELISA kit (DY2780) were from R&D systems Europe. The appropriate capture antibody was diluted in PBS and 100 µL was immediately coated on 96-well microplates, sealed, and incubated at RT overnight. Plates were aspirated and washed three times with PBS containing 0.05% Tween 20. Plates were then blocked with 300 µL 1% BSA in PBS at RT for 1 h. Plates were washed three times with PBS containing 0.05% Tween 20 prior to adding 100 µL of the standard curve or the samples at an appropriate dilution in 1% BSA in PBS. The plates were sealed and incubated for 2 h at RT. The plates were washed with PBS containing 0.05% Tween 20 as previously. The respective HRP-conjugated detection antibodies in 100 µL of 1% BSA in PBS were added to the plate, kept in the dark and incubated for 20 min at RT. The plates were washed with PBS containing 0.05% Tween 20 as above prior to adding 100 µL substrate solution to the wells and a further 20 min incubation. The reaction was terminated using 50 µL 2 M H_2_SO_4_ solution. Plates were read in a FLUOstar Omega plate reader (BMG Labtech) set at 450 nm. Data were analysed using Graphpad Prism.

### 3.5. Secretome Proteomic Analysis

#### TMT Labelling and High pH Reversed-Phase Chromatography

Media samples from DMSO or TT treated cells (four independent repeats) were concentrated to 95 µL using Amicon Ultra 3kDa centrifugal filter units (Millipore, Burlington, MA, USA) and 1 M triethyl ammonium bicarbonate (TEAB) added to achieve a final concentration of 50 mM TEAB. Samples were reduced (10 mM tris(2-carboxyethyl)phosphine (TCEP), 55 °C for 1 h), alkylated (18.75 mM iodoacetamide, room temperature for 30 min.) and then digested with trypsin (2.5 µg trypsin; 37 °C, overnight). The resulting peptides were labelled with Tandem Mass Tag (TMT) ten plex reagents according to the manufacturer’s protocol (Thermo Fisher Scientific) and the labelled samples combined.

The combined sample was desalted using a SepPak cartridge according to the manufacturer’s instructions (Waters, Milford, MA, USA). Eluate from the SepPak cartridge was evaporated to dryness and resuspended in buffer A (20 mM ammonium hydroxide, pH 10) prior to fractionation by high pH reversed-phase chromatography using an Ultimate 3000 liquid chromatography system (Thermo Fisher Scientific). In brief, the sample was loaded onto an XBridge BEH C18 Column (130Å, 3.5 µm, 2.1 mm × 150 mm, Waters, UK) in buffer A and peptides eluted with an increasing gradient of buffer B (20 mM Ammonium Hydroxide in acetonitrile, pH 10) from 0–95% over 60 min. The resulting fractions (4 in total) were evaporated to dryness and resuspended in 1% formic acid prior to analysis by nano-LC MS/MS using an Orbitrap Fusion Tribrid mass spectrometer (Thermo Scientific).

### 3.6. Nano-LC Mass Spectrometry

High pH RP fractions were further fractionated using an Ultimate 3000 nano-LC system in line with an Orbitrap Fusion Tribrid mass spectrometer (Thermo Scientific). In brief, peptides in 1% (*v*/*v*) formic acid were injected onto an Acclaim PepMap C18 nano-trap column (Thermo Scientific). After washing with 0.5% (*v*/*v*) acetonitrile 0.1% (*v*/*v*) formic acid peptides were resolved on a 250 mm × 75 μm Acclaim PepMap C18 reverse phase analytical column (Thermo Scientific) over a 150 min organic gradient, using 7 gradient segments (1–6% solvent B over 1 min., 6–15% B over 58 min., 15–32% B over 58 min, 32–40% B over 5 min, 40–90% B over 1 min, held at 90% B for 6 min and then reduced to 1% B over 1 min) with a flow rate of 300 nl/min. Solvent A was 0.1% formic acid and Solvent B was aqueous 80% acetonitrile in 0.1% formic acid. Peptides were ionised by nano-electrospray ionisation at 2.0 kV using a stainless-steel emitter with an internal diameter of 30 μm (Thermo Scientific) and a capillary temperature of 275 °C. 

All spectra were acquired using an Orbitrap Fusion Tribrid mass spectrometer controlled by Xcalibur 2.1 software (Thermo Scientific) and operated in data-dependent acquisition mode using an SPS-MS3 workflow. FTMS1 spectra were collected at a resolution of 120,000, with an automatic gain control (AGC) target of 200,000 and a max injection time of 50 ms. Precursors were filtered with an intensity threshold of 5000, according to charge state (to include charge states 2–7) and with monoisotopic peak determination set to peptide. Previously interrogated precursors were excluded using a dynamic window (60 s +/− 10 ppm). The MS2 precursors were isolated with a quadrupole isolation window of 1.2 m/z. ITMS2 spectra were collected with an AGC target of 10,000, max injection time of 70 ms and CID collision energy of 35%.

For FTMS3 analysis, the Orbitrap was operated at 50,000 resolution with an AGC target of 50,000 and a max injection time of 105 ms. Precursors were fragmented by high-energy collision dissociation (HCD) at a normalised collision energy of 60% to ensure maximal TMT reporter ion yield. Synchronous precursor selection (SPS) was enabled to include up to 10 MS2 fragment ions in the FTMS3 scan.

### 3.7. Proteomic Data Analysis

The raw data files were processed and quantified using Proteome Discoverer software v2.1 (Thermo Scientific) and searched against the UniProt Human database (downloaded January 2021; 178486 sequences) using the SEQUEST HT algorithm. Peptide precursor mass tolerance was set at 10 ppm, and MS/MS tolerance was set at 0.6 Da. Search criteria included oxidation of methionine (+15.995 Da), acetylation of the protein N-terminus (+42.011 Da) and Methionine loss plus acetylation of the protein N-terminus (−89.03 Da) as variable modifications and carbamidomethylation of cysteine (+57.021 Da) and the addition of the TMT mass tag (+229.163 Da) to peptide N-termini and lysine as fixed modifications. Searches were performed with full tryptic digestion and a maximum of 2 missed cleavages were allowed. The reverse database search option was enabled, and all data were filtered to satisfy the false discovery rate (FDR) of 5%.

### 3.8. Protein Annotation

The MS data were searched against the human Uniprot database retrieved on 2022-01-20, and updated with additional annotation information on 2022-05-13. Protein groupings were determined by PD2.4; however, the master protein selection was improved with an in-house script that identifies identical protein sequences in the uniprot database then selects the accession with the highest annotation quality for use. The script further takes the candidate master proteins for each group, and uses Uniprot review and annotation status to select the best annotated protein as master protein without loss of identification or quantification quality. 

### 3.9. Statistical Analysis

The statistical significance of differences in abundance between control and treated was determined by univariate pairwise t-tests conducted on log2 transformed abundances. Due to the hypothesis-generating nature of the proteomics experiment, FDR correction was not applied, and all candidates were instead confirmed by Western blot analysis [21].

### 3.10. Volcano Plots

For each comparison, using both raw and normalised data, the −log_10_ *p*-value of each protein was plotted against the log_2_ fold change. Proteins with *p* < 0.05 and log^2^ Fold change > 1 changes were annotated in green, and proteins with *p* < 0.05 and Log^2^ Fold change < −1 were highlighted in pink. 

## 4. Results

### 4.1. TT Treatment Leads to MARCKS Phosphorylation on S^167/170^

We initially tested TT dependent phosphorylation of the myristoylated alanine-rich C-kinase substrate, MARCKS, as a downstream target of PKC activation [22] to establish the time frame of TT dependent phosphorylation responses. H357 cells were treated with 1 µg/mL TT for 10 min, 1 h and 24 h or DMSO control. Lysates were analysed by Western blotting and quantified for p-S^167/170^ MARCKS levels. Figure 1C,D shows rapid phosphorylation of MARCKS on S^167/170^ at 10 min and 1 h time points with increases ranging from 16- to 18-fold, respectively, whilst p-S^167/170^ levels were equivalent to the DMSO solvent control at 24 h. Therefore, subsequent experiments were performed at either the 10 min or 1 h time point, unless otherwise stated.

### 4.2. TT Treatment Induces Ser^985^ Phosphorylation of the MET Receptor

**Figure 2:**
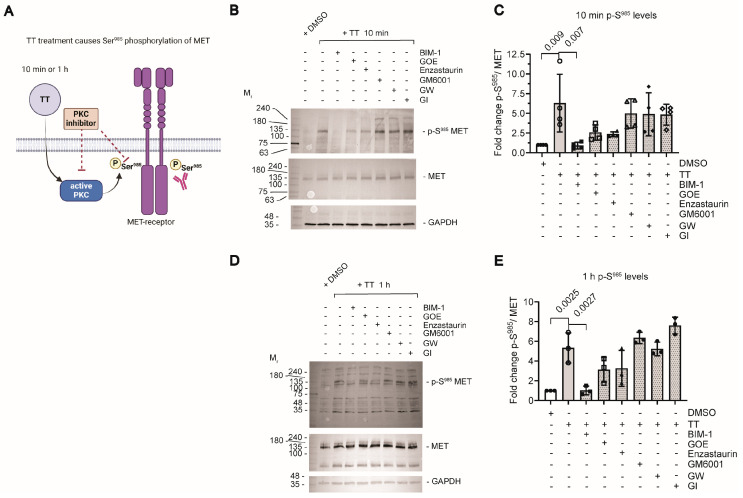
**Determination of p-S^985^ MET phosphorylation levels.** (**A**) Schematic representation of experimental hypothesis prepared in Biorender. (**B**,**D**) Western blot analysis of p-S^985^ MET phosphorylation levels in response to TT treatment in the absence or presence of PKC or metalloproteinase inhibitors. (**C**,**E**) Quantification of p-S^985^ MET phosphorylation levels normalised to the solvent DMSO alone control and total MET levels (*n* = 4 for (**C**) and *n* = 3 for (**E**)). Data were analysed using one way ANOVA with post-Tukey test.

As TT is related to the phorbol ester PMA, we next hypothesised that the drug would be able to induce ectodomain shedding of transmembrane ADAM substrates that require Ser phosphorylation to induce cleavage, as previously shown for neuregulin-1 (NRG1) in response to PMA treatment [23]. We addressed the hypothesis that TT treatment of H357 cells caused PKC dependent changes in the phosphorylation status of the MET receptor on S^985^ (Figure 2A). We pre-incubated cells with 1 μM PKC inhibitors or general metalloproteinase [GM6001 at 25 μM] and ADAM inhibitors [GW and GI at 1 μM] for 1 h, followed by subsequent TT treatment for 10 min or 1 h, which was then compared to DMSO solvent controls in the presence or absence of TT as indicated (Figure 2B–E). Analysis of cell lysates for phosphorylation levels of S^985^ MET was determined by Western blotting (Figure 2B,D; 10 min and 1 h). TT treatment resulted in significant 6-fold or 4-fold increases in full length p-S^985^ MET compared to DMSO controls (Figure 2C,E). Furthermore, pretreatment of cells with the pan-PKC inhibitor BIM-1 ablated S^985^ phosphorylation of full-length MET in response to TT co-treatment (Figure 2B,D). However, the more specific PKC isoform inhibitors GOE and Enzastaurin were less potent and only partially blocked TT dependent S^985^ MET phosphorylation (Figure 2B–E). In contrast, TT-treated samples that were pretreated with metalloproteinase (GM6001, GW or GI) inhibitor showed elevated S^985^ phosphorylation of full-length MET (4–6 fold higher; Figure 2C,E), similar to TT treatment alone. Of note, there were no changes in p-S^985^ MET cleavage products in lysate samples.

### 4.3. Prolonged TT Treatment Reduces Basal Y^1234/5^ Phosphorylation in the Kinase Domain of the MET Receptor in H357 Cells

**Figure 3:**
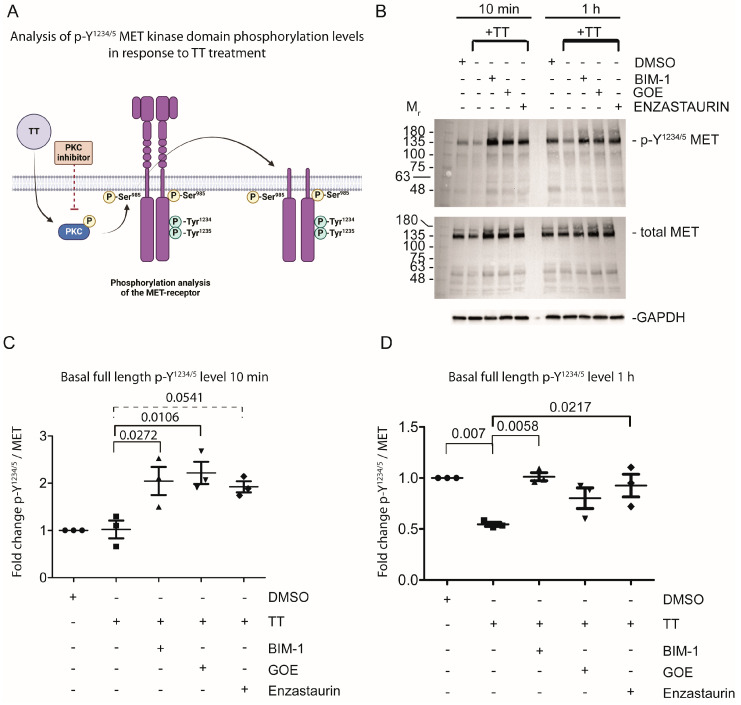
**Prolonged incubation of H357 cells with TT (EBC-46) diminishes basal Y^1234/5^ phosphorylation of the MET receptor.** (**A**) Schematic representation of experimental hypothesis prepared in Biorender. (**B**) Western blot analysis of Y^1234/5^ phosphorylation levels in response to TT treatment in the presence and absence of PKC inhibitor treatment. (**C**,**D**) Fold change of p-Y^1234/5^ levels of full-length MET in response to treatment normalised to solvent control and total MET levels (*n* = 3). Data were analysed using one-way ANOVA with post-Tukey test.

As H357 cells significantly overexpress the MET receptor, we next sought to address the hypothesis that TT treatment would influence basal MET phosphorylation levels on Y^1234/5^, which is an indicator of MET activation status (reviewed by Zhang et al. 2018) [24]. Cells were in part pretreated with PKC inhibitors for 1 h, prior to TT treatment for either 10 min or 1 h to assess the basal phosphorylation status of Y^1234/5^ MET by Western blot analysis (Figure 3B–D). The basal p-Y^1234/5^ levels of full-length MET in response to 10 min TT treatment remained at the same level as the DMSO solvent control (Figure 3B left panel and C quantified), with PKC inhibitor pretreatment and TT stimulation increasing p-Y^1234/5^ levels. In contrast, prolonged treatment with TT for 1 h led to a significant reduction in p-Y^1234/5^ levels of full-length MET, compared to DMSO solvent control or PKC inhibitor treated samples (Figure 3B right panel and D quantified).

### 4.4. Does TT Regulate MET Cell Surface Levels and Cleavage?

**Figure 4:**
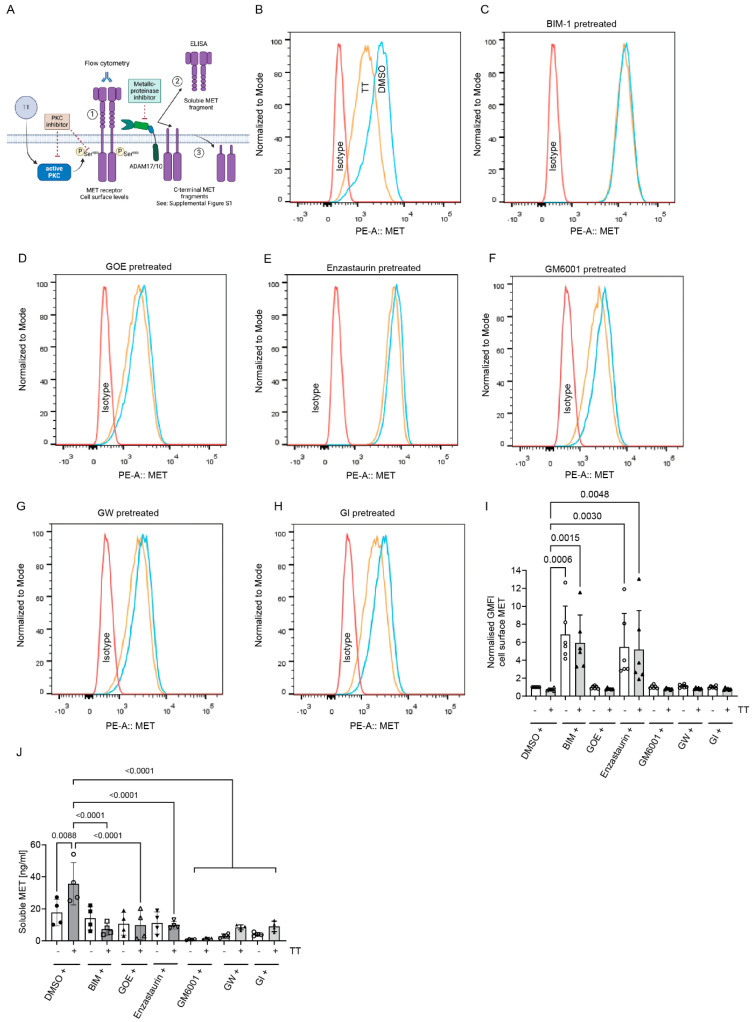
**BIM-1 and Enzastaurin significantly increases cell surface MET levels in H357 cells regardless of TT (EBC-46) treatment, while metalloproteinase inhibitors are ineffective.** (**A**) Schematic representation of experimental hypothesis prepared in Biorender. (**B**–**E**) FACS analysis of cell surface MET levels using an N-terminal MET antibody in DMSO control or PKC inhibitor pre-treated conditions in the presence or absence of TT. (**F**–**H**) FACS analysis of cell surface MET levels in DMSO control or metalloproteinase inhibitor pretreated conditions in the presence or absence of TT. Metalloproteinase inhibitors were GM6001, GW, and GI. Control, blue; TT-treated, orange; and isotype control, red. (**I**) Normalised geometric mean fluorescence levels (GMFI) of cell surface MET (*n* = 3), one way ANOVA with Kruskal–Wallis post-test. (**J**) Analysis of soluble MET levels in conditioned medium following 1 h treatment as described in (**B**–**H**). (*n* = 3), one-way ANOVA with post-Tukey test.

To determine whether the increased S^985^ phosphorylation and loss of Y^1234/5^ phosphorylation on full-length MET in response to TT treatment was associated with the proteolytic cleavage and loss of MET from the cell surface (reviewed in [20,25]) we next determined MET cell surface levels by FACS analysis under both control or PKC inhibitor pretreatment conditions (Figure 4B–F). Flow cytometric analysis showed a trend in reduction of MET cell surface levels in response to TT treatment when compared to DMSO control (Figure 4B), but this was not significant (Figure 4I). However, pre-treatment with BIM-1 caused a significant increase of MET cell surface levels (Figure 4C), confirmed by analysis of the normalised geometric mean fluorescence data (Figure 4I) and was independent of TT co-treatment. Enzastaurin treatment also caused significant increases in surface MET levels regardless of control or TT co-treatment (Figure 4E,I). In contrast, GOE-treated samples with TT showed equivalent cell surface MET levels to the DMSO solvent control (Figure 4D,I). Since MET undergoes proteolytic cleavage in response to PKC activation [20,25,26], we next tested whether metalloproteinase inhibitor pretreatment in the presence or absence of TT could increase MET cell surface levels (Figure 4F–H). The analysis of the metalloproteinase inhibitor-treated samples (GM6001, GW or GI) showed that in control conditions, MET cell surface levels were unaltered, highlighting the limitations of flow analysis when changes are small and adherent cells must be removed from tissue culture plastic prior to analysis. We therefore investigated soluble MET extracellular domain (MET-ECD) levels by ELISA to compensate for the limitation of the flow analysis. This showed a significant increase in soluble MET in response to TT treatment when compared to DMSO control, which was blocked by pretreatment with all PKC inhibitors (Figure 4J). The significant reduction in soluble MET-ECD in media from TT and metalloproteinase inhibitor-treated samples compared to TT alone was also demonstrated. Only GM6001 treatment was successful in significantly suppressing basal MET-ECD release when compared to DMSO solvent control (Figure 4J).

We also analysed C-terminal MET fragment levels using a phosphorylation independent C-terminal MET antibody in cell lysate by Western blotting (Figure S1B). Here, metalloproteinase fragment levels (55 kDa) did not significantly change when compared to DMSO and TT alone controls (Figure S1B,C). However, analysis of γ-secretase fragment levels (50 kDa) revealed a reduction in TT-treated conditions by approximately 50% (Figure S1D). Pretreatment with GM6001 did not alter 50 kDa fragment levels significantly when compared to DMSO or TT treatment. In contrast, GW and GI pre-treatment dramatically reduced γ-secretase fragment levels to <25% of solvent levels, in both the absence and presence of TT. 

**Figure 5:**
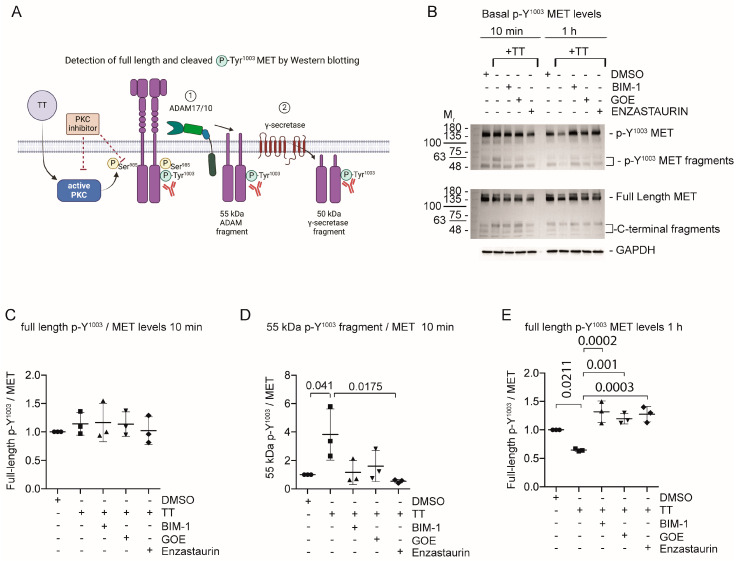
**MET phosphorylated on Y^1003^ is rapidly cleaved in response to TT (EBC-46) treatment.** (**A**) Schematic representation of experimental hypothesis prepared in Biorender. (**B**) Western blot analysis for phosphorylated Y^1003^ full-length MET as well as phosphorylated Y^1003^ MET fragment levels, compared to total MET and fragment levels. (**C**–**E**) Densitometric quantification of full length phosphorylated Y^1003^ MET (10 min) as well as the phosphorylated Y^1003^ 55 kDa ADAM fragment (10 min) or full-length p-Y^1003^ MET (1 h). One-way ANOVA with post-Tukey test.

TT caused rapid S^985^ phosphorylation (Figure 2), which subsequently caused a reduction in active MET phosphorylated at Y^1234/5^ in H357 cells under basal conditions. We were unable to determine significant increases in the 55 kDa MET metalloproteinase fragment using a C-terminal antibody detecting non phosphorylated MET. This is likely due to additional cleavage events that have been described by others (reviewed in [27]). We hypothesised that phosphorylated MET species were the target of metalloproteinase cleavage (Figure 5A). Thus, we next wanted to investigate whether TT treatment also affected the levels of p-Y^1003^ MET. Western blots were stained with an antibody specific for Y^1003^ phosphorylation (Figure 5B–E). As seen in Figure 5B,D rapid formation of the 55 kDa p-Y^1003^ MET fragment was observed at 10 min of TT treatment, which was blocked by all three PKC inhibitors with full length Y^1003^ phosphorylated MET levels and, γ-secretase fragment levels remaining unchanged at 10 min (Figure 5C). 

Prolonged TT treatment for 1 h led to a significant drop in full-length p-Y^1003^ MET (Figure 5B; 1 h panel). At 1 h treatment with TT the 55 kDa p-Y^1003^ MET fragment had been further degraded and was below the detection limit. PKC inhibitor treatment rescued full length p-Y^1003^ MET levels. Additional analysis of the 24 h time point showed a dramatic loss of full-length p-Y^1003^ MET in response to TT treatment, suggesting that the effect of TT on p-Y^1003^ MET was long lasting (Figure S2A,B).

### 4.5. Secretome Analysis of H357 Cell Treated with TT

**Figure 6:**
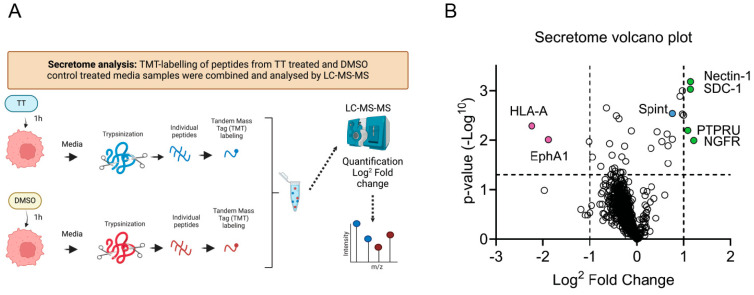
**Secretome analysis of TT (EBC-46) treated H357 cells.** (**A**) Schematic presentation of experimental hypothesis prepared in Biorender; (**B**) Volcano plot of secretome changes. Boundaries of log2 fold changes are indicated by dashed lines (−1 and 1), as well as the −log *p*-value of 0.05.

TT is related to the prototype phorbol ester PMA, which promotes ADAM17 activation [11,28,29,30,31]. We therefore hypothesised that TT could induce proteolytic processing of other transmembrane proteins, leading to the uncloaking of tumour cells so that they become detectable by the immune system, as observed in animal models [1]. We therefore embarked on a proteomic analysis of additional early drug targets using an unbiased proteomic approach. To minimise transcriptional responses to TT treatment, an early 1 h time point was chosen, to determine global secretome changes. This was achieved using TMT labelling and nano-LC mass spectrometry followed by quantification with Proteome Discoverer software, as outlined in methods and Figure 6A. Figure 6B shows a volcano plot with log2 fold changes induced by 1 h TT treatment. Surprisingly, the analysis demonstrated that there was a reduction in proteins released into the cell culture media in TT-treated conditions, when compared to DMSO control. Only four proteins in the secretome were identified to reach the significance threshold and this may indicate the limitations of our analysis at this early 1 h time point. As shown in the volcano plot, TT treatment promoted significant secretion/cleavage of four type I transmembrane proteins into the culture media (Figure 6B). Of these proteins Nectin-1 [12,32] plays roles in cell adhesion, as well as Syndecan-1 [33]; the latter, however, also acts as a co-receptor for signalling molecules, including the MET receptor [34]. Additionally, the neurotrophin receptor NGFR^p75^/TNFR16 [14], which belongs to the tumour necrosis receptor (TNFR) family and the pseudo-receptor phosphatase PTPRU/PCP-2 [35] were above the significance threshold in our secretome analysis. All four proteins have been previously identified as ADAM substrates, supporting our hypothesis that TT regulates ADAM dependent ectodomain shedding in our H357 head and neck cancer cell model [12,32,33,36,37,38,39]. Additionally, TT treatment reduced soluble HLA-A and EphA1 levels significantly in media samples, while Spint/HAI-1, a regulator of MET signalling, was not upregulated (Figure 6B).

**Figure 7:**
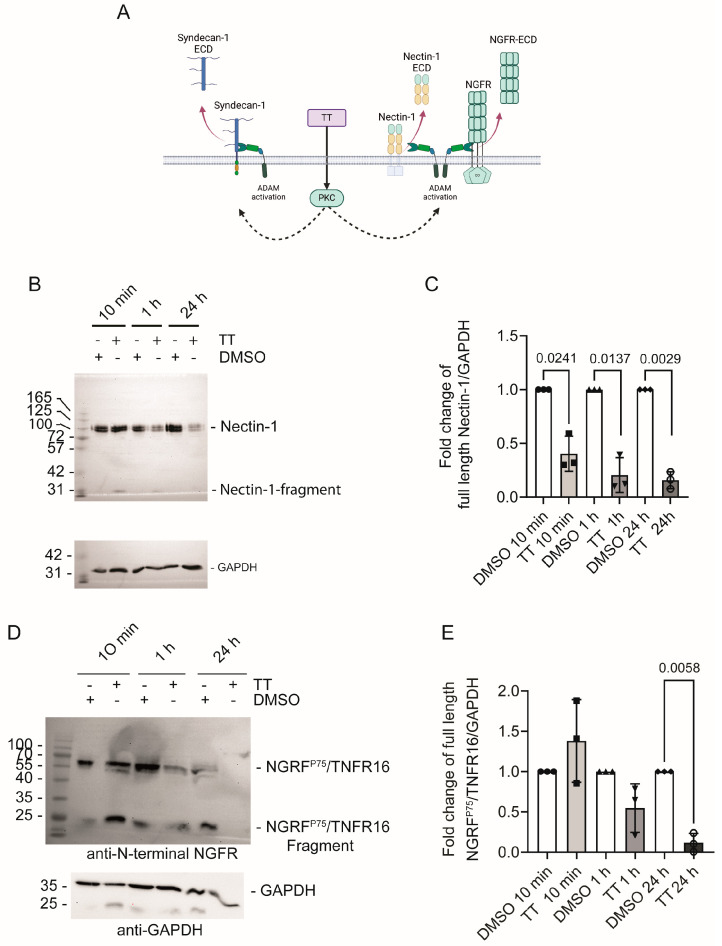
**Nectin-1 and NGFR^p75^/TNFR16 levels’ decline in response to TT (EBC-46) treatment.** (**A**) Schematic presentation of experimental hypothesis prepared in Biorender; (**B**) Western blot analysis of Nectin-1 levels in cell lysates using an N-terminal Nectin-1 antibody; (**C**) Quantification of fold change of full-length Nectin-1 over GAPDH; (**D**) Western blot analysis of NGFR^p75^/TNFR16 levels in cell lysates using an N-terminal NGFR^p75^/TNFR16 antibody; (**E**) Quantification of fold change of full length NGFR^p75^/TNFR16 over GAPDH. Statistical analysis was performed separately for each time point against the normalised DMSO solvent control data using T-test with Welch’s correction (*n* = 3).

**Figure 8:**
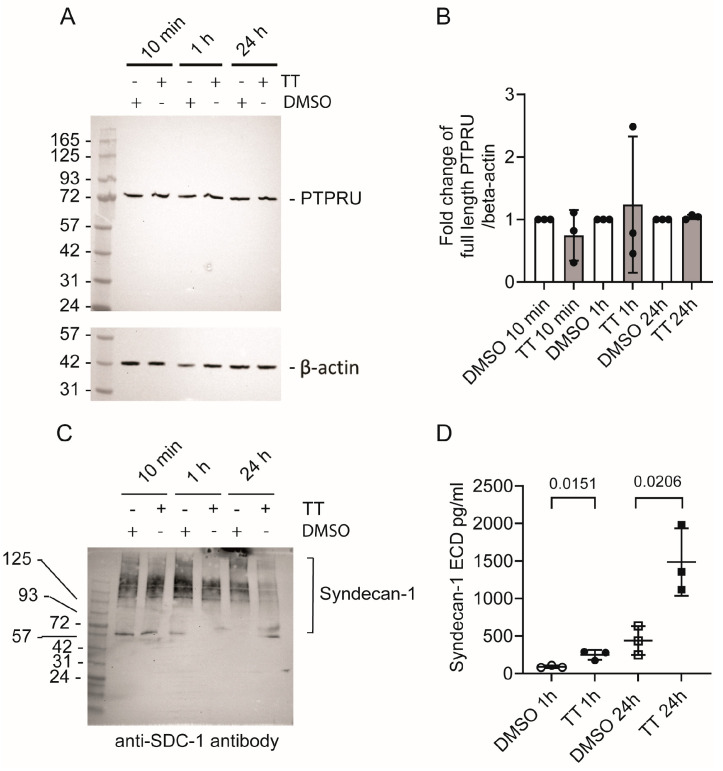
**Analysis of PTPRU and syndecan-1 levels in response to TT (EBC-46) treatment.** (**A**) Western blot analysis of PTPRU levels in cell lysates; (**B**) Quantification of fold change of full-length PTPRU over β-actin (*n* = 3). (**C**) Western blot analysis of syndecan-1 levels in lysate; (**D**) ELISA analysis for soluble syndecan-1-ECD in medium (*n* = 3).

To verify our secretome results, we next performed Western blot analysis of cell lysates from DMSO and TT treated cells, which were probed with Nectin-1 and NGFR^p75^/TNFR16 antibodies recognising the N-terminal domains of these proteins (Figure 8A). Figure 7B shows that TT treatment caused a rapid decline of the 100 kDa Nectin-1 signal in the cell lysate at 10 min, 1 h, and 24 h time points. Quantification of blot signals in the cell lysates to the corresponding GAPDH loading control showed significant Nectin-1 loss from the 10 min timepoint onward (Figure 7C).

Figure 7D shows that the signal for full-length NGFR^p75^/TNFR16 band at 60 kDa rapidly disappeared in response to TT treatment, with complete loss of signal at 24 h. An additional C-terminal cleavage product was visible at 25 kDa at 10 min and 1 h respectively. This 25 kDa NGFR^p75^/TNFR16 fragment disappeared with prolonged incubation time and was below the detection limit at 24 h. This is likely due to additional processing by γ-secretase, as seen by others previously [14]. Quantification of the full-length NGFR^p75^/TNFR16 band showed a significant reduction in NGFR^p75^/TNFR16 levels at the 24 h time point (Figure 7E).

Next, we sought to determine whether PTPRU and syndecan-1 levels were reduced in cell lysates in response to TT treatment. Figure 8A,B show that PTPRU levels were not significantly altered in response to drug treatment, even after prolonged TT treatment for 24 h. When we analysed Syndecan-1 levels in cell lysates by Western blot analysis, there was a reduction in detectable Syndecan-1 at 1 h and 24 h in TT-treated conditions (Figure 8C). However, this could not be quantified as Syndecan-1 signals were apparent over a large range of molecular weights, as expected for this transmembrane proteoglycan. Therefore, we quantified Syndecan-1 ECD levels in the medium using a human Syndecan-1 ELISA (Figure 8D). Syndecan-1 levels increased 2.7-fold at the 1 h time point and 3.4-fold at the 24 h time point, indicating release of Syndecan-1 in response to TT treatment as predicted from the secretome analysis.

## 5. Discussion

This study investigated cell surface changes induced in H357 head and neck cancer cells to TT, focussing specifically on MET receptor S^985^ phosphorylation status, cell surface levels and ectodomain release, in addition to identifying secretome wide alterations identified by using TMT labelling and mass spectrometry approaches. 

Initially MARCKS phosphorylation in response to TT was demonstrated at 10 min and 1 h time points, to establish kinetics of phosphorylation events. Subsequently MET S^985^ phosphorylation was demonstrated following 10 min and 1 h TT treatment, which was inhibited by pretreating cells with the pan-PKC inhibitor BIM-1, although off-target effects of BIM-1 on other kinases cannot be excluded. In contrast, established classical PKC (PKC-α/βI/βII/γ) and PKC βI/βII inhibitors GOE and Enzastaurin respectively were unable to block S^985^ phosphorylation sufficiently. Our data agree with previous work demonstrating PKC-dependent phosphorylation of S^985^ in the intracellular juxta membrane region of MET by the structurally related phorbol ester 12-O-Tetradecanoylphorbol-13-acetate (TPA), oxidative stress or hepatocyte growth factor (HGF) induced MET activation [40]. Consequently, S^985^ phosphorylated MET was largely nonresponsive to HGF mediated cellular responses due to loss of tyrosine phosphorylation in the kinase domain and C-terminal adapter region [40]. This concurs with our findings that full-length p-Y^1234/5^ MET levels were downregulated in response to TT, which was rescued by PKC inhibition. Furthermore, p-Y^1003^ MET underwent proteolytic processing leading to a 55 kDa p-Y^1003^ MET-fragment which was detected at the 10 min time point. However, this 55 kDa p-Y^1003^ MET-fragment was prone to further proteolytic degradation but stable enough for transient detection. We observed the loss of full-length p-P-Y^1003^ over time (1 h/24 h), and also saw a reduction in p-Y^1234/5^ full-length MET (1 h). These residues are part of Exon 14, and previous studies confirm that p-Y^1003^ facilitates proteasome-mediated MET degradation via Cbl association, allowing for negative regulation of MET kinase activity [40,41]. This area contains a caspase 3 processing site, allowing for MET cleavage, and the corresponding caspase 3 cleavage product amplifies cell death through mitochondrial permeabilization [25,42]. 

Cell surface MET levels were significantly upregulated in response to both BIM-1 or Enzastaurin pretreatment in the presence or absence of TT, indicating that PKC activity is the main regulator of MET cell surface levels in H357 cells. Cell surface MET levels were not affected by metalloproteinase inhibitor treatment. TT caused significant increases in soluble MET-ECD, which was suppressed by all PKC inhibitors and all metalloproteinase inhibitors that we investigated. Here, metalloproteinase inhibitors also reduced background shedding of MET-ECD into media. This may indicate that different PKC isoforms regulate surface levels through distinct mechanisms, such as shedding or endocytosis. 

In the context of cancer therapies, TT is unique in that it mainly activates the PKC-βI and PKC-βII isoforms [2]. Interestingly, PKC-βII has been identified as a potential tumour suppressor gene. A heterozygous loss-of-function mutation in the PKC-β gene was identified in colon cancer, and restoration of wild-type enzyme activity decreased anchorage-dependent growth of tumour cells in vitro [43]. Additionally, tumours grew slower when expressing wild-type PKC-βII using a nude mouse model, indicating that loss of PKC-β activity enhances tumorigenesis. This was later linked to changes in insulin growth factor 1 (IGF1) signalling, which is regulated by PKC-βII in colon cancer [44]. These findings may explain the failure of Enzastaurin, a specific PKC-βI/II inhibitor, in clinical trials as reviewed by Bourhill et al. 2017 [44,45]. Furthermore, reduced proteolytic shedding of receptor tyrosine kinases has been identified as a post-translational mechanism of kinase inhibitor resistance [3]. We identified increased MET cell surface levels in response to PKC inhibition by Enzastaurin and BIM-1, and this may in part be causal for the lack of clinical efficacy of Enzastaurin [45]. In contrast, TT causes S^985^ phosphorylation and degradation of p-Y^1003^ MET, loss of full-length p-Y^1234/5^ MET, as well as MET-ECD release and this may explain in part why TT caused cancer regression in certain in vivo tumour models [1,2,4,8]. MET signalling has been identified to drive cancer recurrence as an evasion strategy to HER2 antibodies in breast cancer [19]. Targeting MET, vascular endothelial growth factor receptor (VEGFR) and AXL using cabozantinib resulted in decreased migration, invasion, and proliferation causing apoptotic cell death in naïve and therapy resistant head and neck cancer cells [46]. TT may potentially suppress this evasion strategy in cells expressing wild-type MET, while tumours expressing exon 14 MET deletion mutants may be resistant to TT treatment, and this will require further analysis using exon 14 MET mutant cells. It may also be informative to analyse the TT clinical trial data and stratify patient outcomes according to MET exon 14 status. 

### 5.1. Early Secretome Targets of TT Treatment Identified by TMT Proteomics

Our secretome analysis was performed to identify early TT drug targets that were regulated by proteolysis resulting in the release of cell surface proteins from tumour cells. The 1 h time point was chosen to minimise transcriptional responses due to TT dependent PKC activation. Surprisingly, secretome analysis at 1 h showed that TT treatment had reduced protein levels in culture media when compared to DMSO controls. Analysis of log2 fold changes identified four type I transmembrane proteins that were above the significance threshold. These were Nectin-1, syndecan-1, NGFR^p75^/TNFR16 and PTPRU. These proteins have all been identified as ADAM substrates previously [32,33,38,47]. 

#### 5.1.1. Nectin-1

Nectins are immunoglobulin-like cell adhesion molecules that contribute to the formation of cell–cell adhesions, which regulate cell polarity, movement, proliferation, and survival. Nectins localise to adherence junctions between epithelia and fibroblasts, as well as contributing to tight junction formation in epithelia (reviewed in [48]). Together with Nectin-like molecules, Nectins drive vascular development and barrier function (reviewed in [49]). We here observed rapid Nectin-1 cleavage in response to TT treatment in tumour cells, leading to cell surface loss of Nectin-1. Previous work by Kim et al. showed that Nectin-1 cleavage in neuronal cells depends on ADAM10 and subsequent γ-secretase cleavage [32]. Whether this is the case here requires further analysis. Furthermore, an extracellular Nectin-1/3 fragment was shown to interfere with E-cadherin based adherence junction formation [50]. In the context of TT as an anticancer therapy, we hypothesise that the disruption of tumour tissues and blood supply that was seen in animal models [1,8] may be linked to the ability of TT to disrupt epithelial—and likely also endothelial—Nectin-family members due to irreversible cleavage. Furthermore, in vivo, the intratumoral administration of TT may also affect immune cells, as these interact with Nectin-family members via T-cell activation increased late expression (Tactile or CD96), TIGIT, T-cell immunoglobulin inhibitory and immunoreceptor tyrosine-based inhibitory motif (ITIM) or DNAM1 (CD226) [49]. In this context, cleavage of CD96, TIGIT, or DNAM1 in tumour-infiltrated lymphocytes cannot be excluded in response to TT.

#### 5.1.2. NGFR^p75^/TNFR16

The NGFR^p75^/TNFR16 death receptor has previously been shown to be regulated by differential proteolytic cleavage in hippocampal neurons versus cerebellar neurons [14]. The increased presence of the membrane-associated C-terminal ADAM fragment induced death signalling via caspase-3 in hippocampal neurons, while the intracellular fragment initiated NFκB survival signalling in cerebellar neurons. Therefore, responses are fragment- and cell-type-specific and tightly controlled by proteolysis with γ-secretase cleavage initiating survival signalling. Interestingly, NGFR^p75^/TNFR16 is upregulated in melanoma after both chemotherapy and targeted therapy, and endogenous expression of neurotrophin ligands results in autocrine proliferation and migration [51]. Furthermore, autocrine NGFR^p75^/TNFR16 survival signalling in triple-negative breast cancer is mechanistically linked to NGFR^p75^/TNFR16 overexpression and reduced cleavage [52]. Recent attempts at specific activation of the NGFR^p75^/TNFR16 intracellular domain with a short peptide derived from β-amyloid in combination with MAPK inhibitors or chemotherapy-induced apoptosis in 2D/3D cultures of numerous melanoma cell lines and prevented metastasis in zebrafish xenograft models and reduced tumour mass in mice [53]. TT-dependent NGFR^p75^/TNFR16 cleavage would disrupt neurotrophin dependent proliferation in cancer cells due to receptor shedding, and this may in part contribute to TT having efficiency in melanoma treatment, where four patients reached a complete response [9]. 

#### 5.1.3. PTPRU

The R2B tyrosine phosphatase receptor family (PTPR) consists of four members (PTPRK, PTPRM, PTPRT, and PTPRU) that are characterised by large extracellular domains, which form homophilic interactions. The extracellular domain is linked to a transmembrane helix and to two intracellular phosphatase (PTP) domains. These enzymes regulate spatiotemporal kinase activity, allowing for the fine tuning of tyrosine phosphorylation at the cell membrane as well as intracellularly, following proteolytic processing (reviewed in [47]). However, they also independently regulate cell adhesion as they can interact with cell adhesion regulators. Recently, an elegant study demonstrated that PTPRU is a redox-sensitive pseudophosphatase due to alterations in the membrane proximal phosphatase domain [35]. However, substrate recognition was maintained, allowing PTPRU to compete with the active family members for substrates. Our proteome analysis suggested initially that PTPRU was released into media in response to TT; however, we could not confirm this finding using a Western blot approach, suggesting that PTPRU remains unaltered. 

#### 5.1.4. Syndecan-1

Syndecan-1 is a transmembrane proteoglycan, where the core protein contains glycosaminoglycan chains that are comprised of heparan sulphate and chondroitin sulphate of varying length, which interact with different growth factors. Subsequently, signalling responses with syndecan-1 acting as a co-receptor facilitate fibroblast growth factor, vascular endothelial growth factor, Wnt, as well as HGF signalling to their respective receptors (reviewed in [54]). Syndecan-1 shedding is regulated by metalloproteinases [33,37,38,55]. In the context of our work, syndecan-1 shedding in response to TT may downregulate MET signalling, as this has previously been shown to require cell surface syndecan-1 [34], and we saw loss of p-Y^1234/5^ or p-Y^1003^ MET species in our analysis. High levels of soluble syndecan-1 act as HGF carriers and are capable of inhibiting HGF dependent MET responses [56]; however, in other contexts, they contribute to metastasis (reviewed in [57]). 

### 5.2. Potential Long-Term Implications on Head and Neck Cancer Treatment Strategies

In summary, we demonstrate here that TT regulates MET and NGFR^p75^/TNFR16 signalling, as well as Nectin-1 mediated cell adhesion. These findings may explain in part the efficacy of intratumoural application of TT in animal tumor models. The negative regulation of MET signalling may be of interest in the context of head and neck cancer therapy. In the recent study by Cullen et al. [6], they showed oncolytic tumor disintegration in conjunction with increased caspase activity in response to TT treatment. This could be linked to the MET pathway, as caspase cleavage products of MET cause mitochondrial permeabilisation, which controls the cell death/survival balance [42]. Mitochondrial permeabilisation was one of the major mechanistic findings highlighted by Cullen et al. [6]. Co-treatment with TT and PDL1 checkpoint inhibitor blockers promoted prolonged survival in their animal model [6], suggesting that future therapies may include immune checkpoint blockers in conjunction with TT to promote patient survival. Our study has identified early TT drug responses that mechanistically underpin, and support work performed by others using in vivo mouse tumor models. However, we still lack a comprehensive understanding of the drugs mechanism of action with respect to responses of TT treatment causing changes to the tumor microenvironment. Here cancer associated fibroblasts, T-cells, leucocytes, and endothelial cell responses could all contribute to drug efficacy, which will be the focus of future work. It is hard to predict how future head and neck cancer therapies using TT will develop, as this could be linked to MET mutation status of each patient’s specific tumor and other factors. Patient stratification is imperative to predict treatment outcome; however, the approach of direct tumor injection is advantageous, as it avoids the systemic cytotoxicity encountered with current chemotherapy regiments in clinical use. 

## Data Availability

Data are available from the authors on request.

## Supplementary Materials

The following supporting information can be downloaded at: https://www.mdpi.com/article/10.3390/cells13110982/s1, Figure S1: Western blot analysis of non-phosphorylated C-terminal fragment formation using a C-terminal MET antibody. (A) Schematic representation of experimental approach. (B) Western blot analysis. (C) Quantification of the 55 kDa ADAM fragment. (D) Quantification of γ-secretase fragment levels. Figure S2: Western blot analysis of p-Y^1003^ MET levels in H357 cell lysates. (A) Western blot analysis. (B) Quantification of blots for full length p-Y^1003^ MET. Figure S3: Gating strategy for flow cytometry analysis on cell surface MET (A) H357 cells gating, Side scatter area (SSC-A) vs. Forward scatter area (FSC-A) (B) Single cells, Forward scatter height (FSC-H) vs. FSC-A (C) Single cells, Forward scatter width (FSC-W) vs. FSC-A (D) Live/Dead (LD) gating, LD gating (E) Isotype control histogram and c-MET gating. Flow cytometry was done on BD Canto II flow cytometer. Analysis was performed on Flowjo software.
