# Supplementary material for: Tigilanol Tiglate-Induced Changes in Secretome Profiles Alter C-Met Phosphorylation and Cell Surface Protein Expression in H357 Head and Neck Cancer Cells
Source: Cells. 2024 Jun 5;13(11):982. doi: 10.3390/cells13110982 (PMC11171882; doi:10.3390/cells13110982)
Supplement: Supplementary file 1 [file cells-13-00982-s001.zip › cells-3027541-SI.pdf]

Supplemental information Antwi et al 2024 submission to Cells

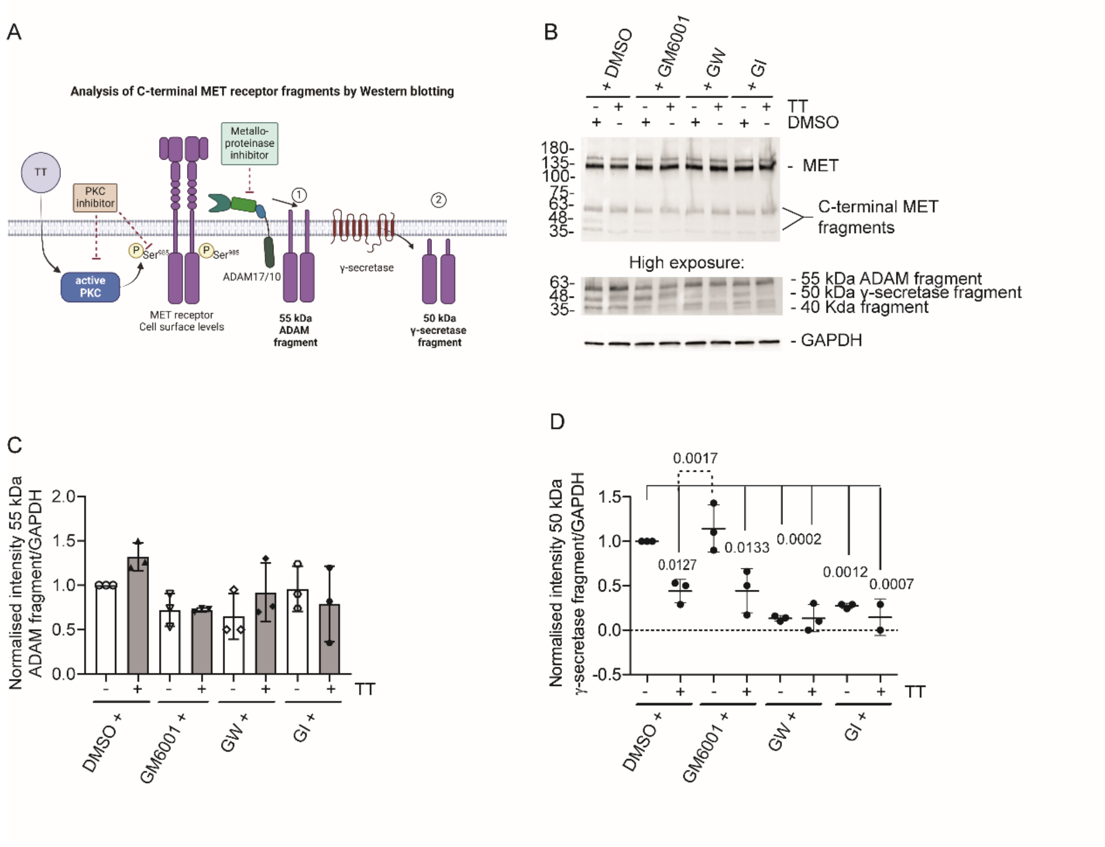

Supplemental Figure S1: Western blot analysis of non-phosphorylated C-terminal fragment formation using a C-terminal MET antibody. (A) Schematic representation of experimental approach. **(B)** Western blot analysis. **(C)** Quantification of the 55 kDa ADAM fragment. **(D)** Quantification of γ-secretase fragment levels.

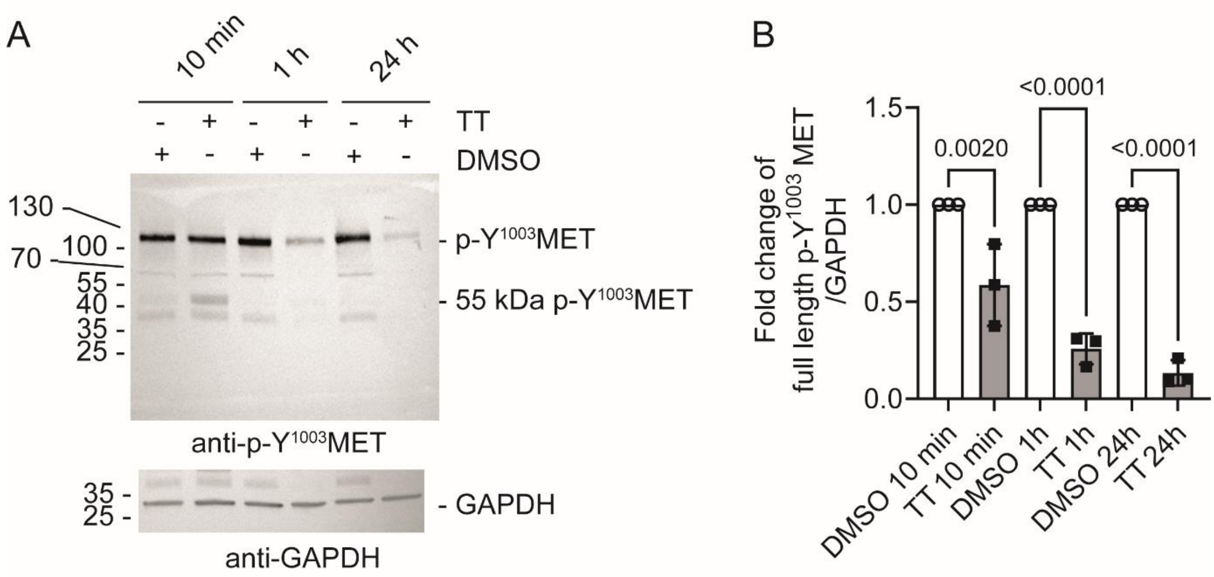

Supplemental Figure S2: Western blot analysis of p-Y^{1003} MET levels in H357 cell lysates. **(A)** Western blot analysis. **(B)** Quantification of blots for full length p-Y^{1003} MET.

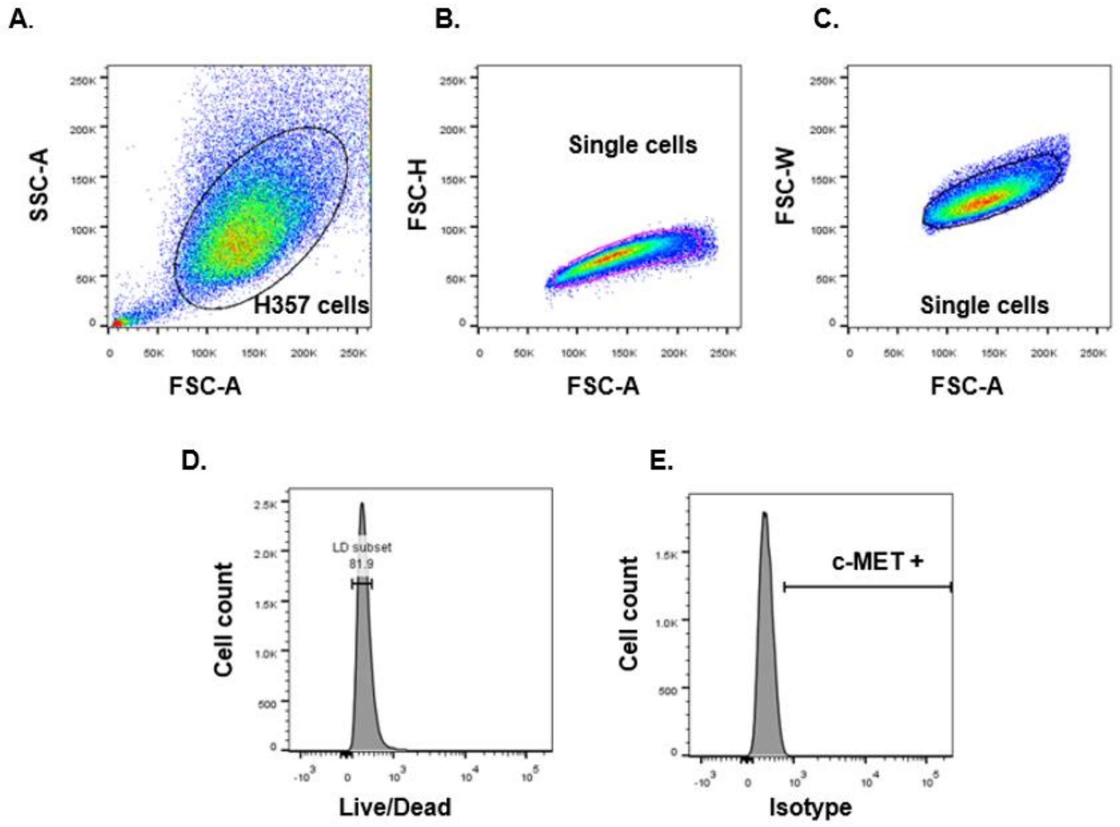

Supplemental Figure S3: Gating strategy for flow cytometry analysis on cell surface MET

(A) H357 cells gating, Side scatter area (SSC-A) vs Forward scatter area (FSC-A) **(B)** Single cells, Forward scatter height (FSC-H) vs FSC-A **(C)** Single cells, Forward scatter width (FSC-W) vs FSC-A **(D)** Live/Dead (LD) gating, LD gating **(E)** Isotype control histogram and c-MET gating. Flow cytometry was done on BD Canto II flow cytometer. Analysis was performed on Flowjo software.
